# Retraction Note: BRCA1 inhibits AR–mediated proliferation of breast cancer cells through the activation of SIRT1

**DOI:** 10.1038/s41598-026-35495-5

**Published:** 2026-01-13

**Authors:** Wenwen Zhang, Jiayan Luo, Fang Yang, Yucai Wang, Yongmei Yin, Anders Strom, Jan Åke Gustafsson, Xiaoxiang Guan

**Affiliations:** 1 Department of Medical Oncology, Jinling Hospital, Medical School of Nanjing University, Nanjing, 210002 China; 2 Department of Medicine, Rutgers New Jersey Medical School, Newark, NJ 07103 USA; 3 Department of Oncology, The First Affiliated Hospital of Nanjing Medical University, Nanjing, 210029 China; 4 Department of Biology and Biochemistry, Center for Nuclear Receptors and Cell Signaling, University of Houston, Houston, TX 77204 USA

Retraction of: *Scientific Reports* 10.1038/srep22034, published online 23 February 2016

The Editors have retracted this Article. After publication, concerns were raised regarding similarities in the blots presented in the Figures, specifically:

- Fig. 1F MCF-7 and T47D GAPDH blots appear highly similar Fig. 2H MCF-7 GAPDH lanes 2-3 and MDA-MB-157 GAPDH lanes 1-2, respectively;
- Fig. 1F MCF-7 BRCA1 blot appears highly similar to Fig. 2C MAC-7 BRCA1 in [1];
- Fig. 1F MCF-7 AR blot appears highly similar to MDA-MB-157 AR (with different exposure);
- Fig. 1F SK-BR-3 BRCA1 lane 2 appears highly similar to Fig. 3D SK-BR-1 mTOR lane 1;
- Fig. 2A T47D Skp2/b lane 2 appears highly similar to Fig. 2F MDA-MB-157 Skp2/b lane 2, flipped horizontally;
- Fig. 2A MDA-MB-157 Skp2/b lane 1 appears highly similar to Fig. 2F MDA-MB-157 Skp2/b lane 1, flipped horizontally;
- Fig. 2F T47D AR blot appears highly similar to Fig. 3F MDA-MB-157 SIRT1 lanes 2-3.

The Editors requested the raw original data underlying the paper, but the Authors were not able to provide it. The Editors therefore no longer have confidence in the presented data.

None of the Authors responded to the correspondence from the Editors about this retraction.
